# Polymorphic analysis of MHClinked Heat Shock Protein 70 genes: Their susceptibility and prognostic implication in Kangri cancer cases of Kashmiri population

**DOI:** 10.4103/0971-6866.55218

**Published:** 2009

**Authors:** Shakeel ul Rehman, A. Syed Sameer, Lubna Zahoor, Nidda Syeed, Mahoor S. Nanda, Adil Hafiz, Zaffar A. Shah, Mushtaq A. Siddiqi

**Affiliations:** Departments of Immunology and Molecular Medicine, Sher-i-Kashmir Institute of Medical Sciences, Soura, Srinagar, Kashmir - 190 011, India; 1 Departments of Plastic Surgery, Sher-i-Kashmir Institute of Medical Sciences, Soura, Srinagar, Kashmir - 190 011, India

**Keywords:** HSPs, mutation, Kangri cancer, Kashmir, RFLP

## Abstract

Kangri cancer is a unique thermally-induced squamous cell carcinoma (SCC) of skin that develops due to persistent use of Kangri (a brazier), used by Kashmiri people, to combat the chilling cold during winter months. We designed a large scale case-control study to characterize the frequency of two polymorphisms within the MHC class III-linked HSP70genes, Hsp70-2 and Hsp70-hom, in order to find any association of these genotypic variants for predisposition to and clinical outcome of Kangri cancer patients from Kashmir valley in North India. Polymerase Chain Reaction and restriction enzymes were utilized to characterize the frequency of two polymorphisms with in Hsp70-2 and Hsp70-hom genes in 118 Kangri carcinoma cases and 95 healthy controls from the same population of Kashmir. Association of high frequency allelic variants of Hsp70genes with various clinicopathological features of prognostic significance was assessed by Chi-square test using SPSS software. In this study, allelic frequency of Hsp70-2 A/G heterozygote (0.87) (*P* = 0.012) was found to be significantly high in Kangri cancer cases compared to control (0.736) with a Relative Risk of 2.45 fold. Conversely, the allelic frequency of Hsp70-2 A/A allele in homozygous condition was significantly low in Kangri cancer cases and worked out to be 0.084 (Vs 0.252 in control) with P is equal to 0.001, implicating it as a protective allele against Kangri cancer in subjects with this genotype. Similarly, significantly high frequency of 0.50 (Vs 0.29 in control) of Hsp70-homC/C allele was found in homozygous condition in Kangri cancer cases suggestive of a positive relative risk associated with this genotype (RR is equal to 2.47) (*P* is equal to 0.002). The overall allele frequency data analysis of Hsp70-2 and Hsp70-hom genes was significant (χ^2^ is equal to 12.38, *P* is equal to 0.002; and χ^2^ is equal to 12.21, P is equal to 0.002). The study also reveals considerable association of high frequency alleles of HSP70 genes, especially of Hsp70-2 A/G or G/G in Kangri tumors with clinico-pathological features of poor prognosis. These results indicate that the relative risk of Kangri cancer associated with Hsp70-2 and Hsp70- hom gene polymorphisms is confined to Hsp70-2 A/G or G/G and Hsp70homC/C haplotype in our population. The study, therefore, suggests Hsp70-2 A/G or G/G and Hsp70homC/C genotypes as potential susceptibility markers and independent prognostic indicators in Kangri carcinoma patients in Kashmiri population.

## Introduction

Kangri cancer represents a unique thermally induced squamous cell carcinoma (SCC) of skin that develops due to chronic irritation by constant exposure to Kangri; the heat pot used to keep body warm during the cold temperatur.[1‐3] The induction of HSP's, occur in wide range of tumors and the mechanism of increased transcription is by either reversal of repression of HSP gene promoter by mutant *TP53* (wild type *TP53* act as repressor of HSP70gene) [4‐6] by increased transcription and stabilization of Heat Shock Factor1 {HSF1}[7] and by positive regulation of HSP genes by protooncogenes like *c-myc*.[8] The 70Kdal HSP70 family includes three intronless genes, Hsp70-1, Hsp70-2, Hsp 70-hom, that have been mapped within the Class III region of MHC complex on 6p21.3.[9,10] Hsp70-1 and Hsp70-2genes are 12kb apart and lie 92kb telomeric to C2gene whileas Hsp70-hom is located 4kb telomeric to Hsp70-1.[9,10] Hsp70-1 and Hsp70-2 encode identical 641 aminoacid proteins[11] whileas the Hsp70-hom encode a 641 aminoacid protein that shares a 90% sequence identity with other Hsp70 proteins.[12] Genetic polymorphism in HSP70genes may influence its anti-apoptotic and immune modulator function and, therefore, may have consequences on predisposition to and prognosis of the disease. This work is a case-control study to investigate a potential association of genetic variation of Hsp70-2 and Hsp70-hom genes with the risk to and prognosis of disease (disease outcome) in a cohort of Kangri cancer cases from Kashmir, North India. Our data indicate that genetic polymorphism in Hsp70-2 and Hsp70-hom genes may represent susceptibility and prognostic indicators.

## Materials and Methods

### Patients and controls

The gene and allele frequencies of the Hsp70-2 and Hsp70-hom genes were determined in a group of 95 control subjects and 118 patients with Kangri cancer. The patients (80 males and 38 females) had been admitted to the Medical Oncology department of Sher-I-Kashmir Institute of Medical Sciences (SKIMS) from 2001-2006. The mean age of patients was 50.7 years. (52.14%) of the cancer patients have ulcerative form, (30.62%) as nodular form, and (17.24%) as fungative form. Most of the patients (93.40%) have metastases in the groin and (5.60%) have axillary metastases. Lymph nodes were involved in (72.03%) and (9.3%) of the cases develop secondaries to muscle periosteum and even bone. The clinical diagnoses were confirmed by histopathological examination, majority of which were of grade II (0.57%). Clinico-pathological data of 118 Kangri cancer patients were collected from the department of Medical Oncology and Pathology of SKIMS, Kashmir, India. Control subjects were healthy blood donors from the same population of Kashmir, who have also used Kangri throughout their life but did not develop the cancer. Written informed consent was obtained from all subjects before taking the sample.

### DNA extraction

Genomic DNA was extracted from peripheral blood leukocytes by standard procedure. About 5 ml of heparinised blood was mixed with 15 ml of DNA lysis solution (155 mM NH_4_Cl, 10 ml KHCO_3_,0.1 mM EDTA; PH 8.0). Leucocytes were spun down; suspended in 10 ml of saline EDTA solution (75 mM NaCl, 20 mM EDTA PH 8.0),1 ml of 10% SDS, and incubated with proteinase K at 37°C in a water bath overnight. DNA was subsequently separated from proteins by phenol chloroform iso-amyl alcohol procedure. DNA in the supernatant fluid was precipitated with ethanol and the pellet was dissolved in 400 μl of DNA storage buffer and stored at 4°C.

### Polymorphism analysis of the Hsp70-2 and Hsp70-hom genes

Polymorphism within Hsp70-2 and Hsp70-hom genes has been characterized by Milner and Campbell (1992) who identified a polymorphic Pst1 site at position 1267A greater than G [=1249A greater than G, (GI is equal to 5123454)] of the Hsp70-2gene and a polymorphic Nco1 site at position 2437(C greater than G) [=1630 C greater than G,(G1I is equal to 27436929)] of the Hsp70-hom gene. The position 1267 of the Hsp70-2gene lies in the coding region, but corresponds to a silent mutation. The polymorphic nucleotide 2437 of the Hsp70-hom gene corresponds to a Met greater than Thr aminoacid substitution. The coding sequence of this Hsp70-2 and Hsp70-hom genes were amplified from g-DNA using sequence specific oligo-nucleotide primers. For Hsp70-2, the 5' primer: 5' ACC CTG GAG CCC GTG GAG AA was used in combination with the 3' primer: 5' CAC CCG CCC GCC CCG TAG G. For Hsp70-hom, the 5' primer: GGA CAA GTC TGA GAA GGT ACA G-3' was used in combination with the 3' primer: 5' GTA ACT TAG ATT CAG GTC TGG 3'. The PCR mixture contained to a final concentration of 500 nano-grams of g-DNA, 200 μM of dNTP's, 1.5 mM MgCl_2_ , 1X Taq polymerase buffer, 10 picomoles/ul of each primer and 1U of Taq polymerase. Amplification was accomplished by initial incubation at 94°C for five minutes followed by 35 cycles of incubation at 94°C for 30 sec; annealing at 62°C for 35sec and 58°C for 45sec respectively for Hsp70-2 and Hsp-hom; and extension at 72°C for 55 sec. followed by final incubation at 72°C for seven minutes. To assess the polymorphism of the Hsp70-2 at position 1267 and that of Hsp-hom at position 2437, the corresponding PCR products were digested with Pst1 (^CTGCA↓G;^ _G_^↑^_ACGCT_) and Nco1 (^C↓CATGG;^ _GGTAC_^↑^_C_) (Restriction enzymes, Fermentas) respectively. The presence of Pst1 in Hsp70-2gene was indicated by the cleavage of the 189bp amplified PCR product to yield fragments of 116bp and 73bp products. The two allelic forms of Hsp70-2 corresponding to the presence and absence of Pst1 site are referred to as Hsp70-2 A/A and Hsp70-2 G/G allele respectively. Similarly, the presence of Nco1 site in Hsp70-hom gene was indicated by the cleavage of 878bp amplified PCR product to yield fragments of 551bp and 327bp. The two allelic forms of Hsp70-hom corresponding to presence and absence of Nco1 site are referred to as Hsp70-hom C/C and Hsp70-homG/G allele respectively.

### Statistical analysis

All statistical analysis was performed using S-PLUS soft-ware. Chi-square test was used to test for a significant association between Kangri cancer and Hsp70-2 and Hsp70-hom genotypes (allelic frequencies). Relative risk associated with a particular genotype was estimated by the odds ratio formula.[13] Chi-square test was used to find any significant association of high frequency alleles in Kangri cancer cases with various clinico-pathological parameters of prognostic significance. The parameters included were Age; less than 50years Vs greater than/equal to50 years; Clinical tumor Stage [T1(a,b)] Vs [T2(a,b)] (TNM);[14] lymph node status (N_1_ Vs N_0_) and histopathological Grade [Grade I Vs (Grade II)].[15] The level of significance was set at *P* less than/equalto 0.05.

## Results

### Polymorphism in Hsp70-2 and Hsp-hom genes as risk factors for Kangri carcin oma

#### (a) HSP70-2 Polymorphism

**Figure 1:**
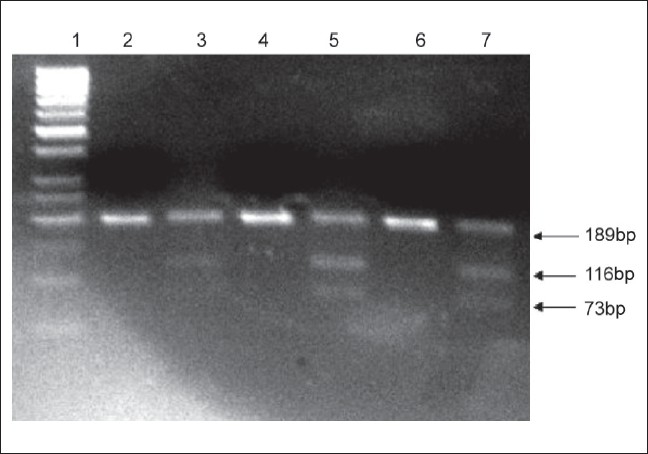
Restriction fragment length polymorphism analysis of Hsp70-2 gene: The Hsp70-2 PCR products(189bp) were digested with Pst1. The Hsp70-2A/Aallele corresponds to 116 and 73bp cleavage products (presence of Pst1 site) and hsp70-2G/G allele corresponds to 189bp uncleaved product (absence of Pst1 site). The reaction products electrophoresed on 3% agarose gel are shown. Lane#1: 50bp Molecular Weight Marker; Lane# 2, 4, 6: undigested HSP 70.2 189bp PCR product; Lane # 3, 5, 7 = Restriction enzyme Pst1 digested HSP 70.2 PCR product (all showing heterozygous change, hsp70-2A/G)

The genotype frequencies of Hsp70-2 in patients with Kangri cancer and the control group from the population of Kashmir is shown in Table 1 [Figure 1].The frequency of Hsp70-2A/A allele in patients with Kangri cancer is 0.084 (Vs 0.252 in controls) resulting in a significantly negative relative risk (RR is equal to 0.273) associated with this genotype (*P* less than 0.05). An increase in frequency of Hsp70-2 A/G heterozygote was observed in Kangri cancer cases (0.87) compared to control group (0.736).The allelic frequency of Hsp70-2 A/G heterozygote was significantly more with a relative risk 2.45 fold in Kangri cancer cases (*P* less than 0.05) compared to control. Conversely the allelic frequency of Hsp70-2 G/G allele was 0.042 in Kangri cancer cases (Vs 0.010 in control) with RR is equal to 4.15. The overall frequency of Hsp70-2 G/G allele in homozygous or heterozygous condition was 0.477 in Kangri cancer cases (Vs 0.378 in control). These results indicate that the RR of Kangri cancer associated with ***Hsp70-2 polymorphism*** is confined to Hsp70-2 G allele in homozygous or heterozygous state while as Hsp70-2 A allele in homozygous condition is rather a protective allele for Kangri cancer in our population.

**Table 1:** Genotype frequencies of Hsp70-2 in control subjects and in patients with Kangri cancer

Genotype	Kangri cancer (n=118) (f)	Control (n=95) (f)	Odd's ratio; χ^2^*P*
Hsp70-2 A/A	(10) 0.08	(24) 0.25	0.273 (95% CI= 0.12-0.60); χ^2^*P* < 0.05
Hsp70-2 A/G	(103) 0.88	(70) 0.74	2.45 (95% CI= 1.20-4.98); χ^2^*P* < 0.05
Hsp70-2 G/G	(5) 0.04	(1) 0.01	$NS; χ^2^ = 12.387, *P* = 0.002

**Allele**		**Allele frequency**	

A	0.52	0.62	0.52 (95% CI= 0.35-0.78); χ^2^*P* < 0.05
G	0.48	0.38	1.505 (95% CI= 1.82-2.22); χ^2^*P* < 0.05

Hsp= Heat shock protein; f = genotype frequencies; χ^2^ = Chi-square; OR= odds ratio;

$ NS= Not significant; The Chi-square test was used whether significant differences (*P*-value) in genotype frequencies were observed when patient group was compared with control subject

#### (b) Hsp70-hom polymorphism

**Figure 2:**
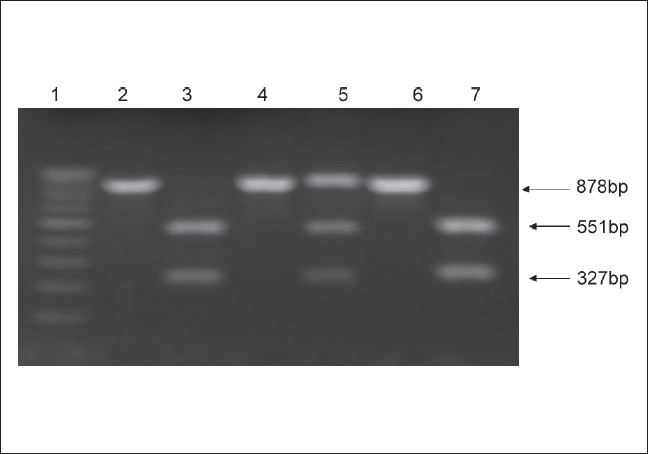
Restriction fragment length polymorphism analysis of hsp70-hom gene: The hsp70-hom PCR products (878bp) were digested with Nco1. The hsp70-homC/C allele corresponds to 551bp and 327bp cleavage products (presence of Nco1 site) and hsp70-hom G/G allele corresponds to 878bp uncleaved product. The reaction products electrophoresed on 1.5% agarose gel are shown. Lane# 1: 100bp Molecular Marker; Lane# 2, 4, 6: undigested HSP70-hom 878bp PCR product; Lane# 3 & 7: Nco1 digested HSP70-hom PCR product (hsp70-homC/C allele); Lane# 5: Nco1 digested HSP70-hom PCR product (hsp70-hom C/G allele)

The allelic frequency of Hsp70-hom genotypes in Kangri cancer patients and control group given in Table 2 [Figure 2] reveal high frequency of Hsp70- hom CC allele in homozygous condition in Kangri cancer cases 0.50 (Vs 0.29 in control) with a significantly positive relative risk associated with this genotype (RR is equal to 2.47) (*P* ≤ 0.05). Conversely, the significantly low frequency of Hsp-homGG genotype in homozygous (0.016) or heterozygous condition (0.474) in Kangri cancer cases compared to control (0.073) and 0.631 respectively) suggest it rather a protective allele for Kangri cancer with negative relative risk (RR is equal to 0.217 ; RR is equal to 0.52 , respectively).These results indicate the relative risk of Kangri cancer associated with Hsp70-hom polymorphism is confined to Hsp70hom CC genotype. The overall Chi-square test used for comparative evaluation of Hsp70-2 and Hsp70-hom gene polymorphic analysis reveals that the data is quite significant (χ^2^ is equal to 12.38, *P* is equal to 0.002; and χ^2^ is equal to 12.21, *P* is equal to 0.002).

**Table 2:** Genotype frequencies of Hsp70-hom in control subjects and in patients with Kangri cancer

Genotype	Kangri cancer (n=118) (f)	Control (n=95) (f)	Odd's ratio; χ^2^*P*
Hsp70-2 C/C	(60)0.51	(28)0.29	2.47(95% CI= 1.43-4.30); χ^2^*P*< 0.05
Hsp70-2 C/G	(56)0.47	(60)0.63	0.526(95% CI= 0.30-0.91); χ^2^*P*< 0.05
Hsp70-2 G/G	(2)0.02	(7)0.08	$NS; χ^2^=12.21, *P* = 0.002

**Allele**		**Allele frequency**	

C	0.74	0.61	1.87(95% CI= 1.23-2.82); χ^2^*P*< 0.05
G	0.26	0.39	0.53(95% CI= 0.35-0.80); χ^2^*P*< 0.05

Hsp= Heat shock protein; f = genotypefrequencies; χ^2^ = Chi-square; OR= odds ratio;

$ NS= Not significant; The Chi-square test was used whether significant differences (*P*-value) in genotype frequencies were observed when patient group was compared with control subjects

The presence of high frequency allelic variants of HSP70's (Hsp70-2 A/G or G/G and Hsp70-hom CC in our population) when compared with various clinico-pathological attributes of Kangri cancer patients showed statistically significant association of Hsp70-2 A/G or G/G genotype with advanced Clinical Tumor Stage [T2 (a,b)] (*P* ≤ 0.05) and histopathological Grade II (*P* ≤ 0.05), features that reflect poor prognosis [Table 3]. However, Hsp70-homCC genotype, though found at high frequency among Kangri cancer cases, was significantly associated with early Clinical tumor stage T1 (a,b) (*P* ≤ 0.05) and histopathological grade I (*P* ≤ 0.05) [Table 3], which suggest it rather a very low risk imposing genotypic variant of Hsp70-hom for Kangri cancer.

**Table 3:** Association of high frequency Hsp70-2A/G or G/G and Hsp70-homC/C genotypes with various clinico-pathological features of Kangri cancer patients of Kashmir

Features	Hsp70-2A/G or G/G		Hsp70-homC/C	
	Genotype (%)	Odd's ratio; χ^2^*P*	Genotype (%)	Odd's ratio; χ^2^*P*
Age		0.116(95%CI=0.02-0.48) χ^2^*P* <0.05		$NS
<50	23/30 (76.66)		17/30 (56.66)	
≥ 50	85/88 (96.59)		43/88 (48.86)	
Clinical tumor stage^a^		ΦNA		3.8(95%CI=1.77-8.14)χ^2^*P*<0.05
T1 (a,b)	50/60 (83.33)		40/60 (66.66)	
T2 (a,b)	58/58 (100)		20/58 (34.48)	
Histopathological grading^b^		ΦNA		3.43(95%CI=1.59-7.41)χ^2^*P*<0.05
Grade I	40/50 (80.00)		34/50 (68.00)	
Grade II	68/68 (100)		26/68 (38.23)	
Lymph node status^c^		7.35(95%CI=1.77-30.52)χ^2^*P*<0.05		0.06(95%CI=0.02-0.21)χ^2^*P*<0.05
N1	82/85 (96.47)		31/85 (35.63)	
N0	26/33 (78.78)		29/33 (88.88)	

Hsp= Heat shock protein; f = genotype frequencies; χ^2^ = Chi–square; OR= odds ratio;

$ NS= Not Significant

Φ NA = Not Applicable; The Chi-square test was used whether significant differences (*P*-value) in genotype frequencies were observed when patient group was compared with control subjects

a Clinical tumor stage: T1 (a,b)= when tumor size ranges from >2 and >5 cm and metastasis to inguinal nodes but no metastasis, T2 (a,b)= tumor of any size, tumor extends to bone, any no of nodes involved and metastasis

b Histopathological tumor grade (TNM classification): Determined based on pathological examination.

c Lymph node status: N1= involved, N0= not involved

## Discussion

Several studies have shown statistical evidence of association between specific human leukocyte antigen (HLA) alleles and risk for or protection against various cancers.[16,17] In the present study we report a strong association between specific Hsp70-2 and Hsp70-hom allelic variants and risk for or protection against Kangri cancers in Kashmiri population.

Comparison of Hsp70-2 allele frequencies in patients with Kangri cancer and control subjects from same population of Kashmir indicated a significant decrease of Hsp70-2 A/A allele in Kangri cancer cases, suggesting it rather a protective allele in homozygous state (*P* ≤ 0.05). Conversely, a high relative risk was found in Kangri cancer cases that carry Hsp70-2 G allele in heterozygous or homozygous state [Table 1]. Our finding is in agreement with the two separate reports from Tunisia, where also statistically significant breast cancer cases carry Hsp70-2 G allele in homozygous state (0.25) (Vs 0.02 in control) (RR =16.3, *P* = 0.0001[18] and 0.280 Vs 0.05 in control (RR = 7.12; *P* is = 0.0001).[19] The Hsp70-2 G allele in homozygous form has been also reported to impart risk in cancers other than Breast like Non-Hodgkins lymphoma (RR = 18.2; *P* = 0.0001),[18] nasopharyngeal carcinoma (RR = 2.309; *P* = 0.006) and in diseases other than cancer.[20‐22]

Similarly, a striking difference in the frequency of Hsp70-hom genotypes was found in Kangri cancer patients of Kashmir when compared with controls [Table 2]. Hsp70-hom C/C genotype in Kangri cancer cases (0.50) (Vs 0.29 in controls) was found to impart significantly high relative risk to Kangri cancer (RR = 2.47, *P* ≤ 0.05) and the risk decreases significantly as the allelic frequency of Hsp70-homG allele increases in homozygous or heterozygous state (RR = 0.52, *P* ≤ 0.05).This is contrary to what has been reported from Tunisia where allelic frequency of Hsp70-hom G allele in homozygous or heterozygous is more in breast cancer cases (0.13) compared to controls (0.05) (RR = 3.4, *P* = 0.01).[18] More interestingly, Hsp70-2 A/A and Hsp70-hom G/G genotypes were found in negligible frequencies in Kangri cancer cases from our Kashmiri population hence are protective alleles to our population.

Comparison between the Hsp70-2 and Hsp70-hom haplotype frequencies in patients and in control subjects indicate almost double frequency of Kangri cancer cases (48/114) (0.42) harbour together Hsp70-2 A/G or GG and Hsp70-hom C/C haplotype when compared with controls (18/90) (0.20). These results suggest that Hsp70-2A/G or G/G and Hsp70-homC/C haplotype may represent a specific risk factor for Kangri cancer to our population of Kashmir.

The polymorphic Pst1 site in Hsp70-2, although a synonymous variation, has functional significance as per recent reports which show the influence of synonymous gene variation on the expression levels and enzyme activity, possibly, by affecting the secondary structure of mRNA, its stability and the timing of co-translational folding that alters the substrate or inhibitor binding sites.[23,24] NcoI Hsp70-hom polymorphism corresponds to Met greater than Thr substitution at amino acid 493, which from a part of peptide binding domain of Hsp70-hom protein.[12] Based on the Hsp-70 structural model, a Met to Thr substitution at amino acid 493 could be associated with variation in peptide binding specificity of Hsp70-hom between haplotypes. The variation may influence antigen presentation of Hsp70-hom of tumor derived antigens to cytotoxic T lymphocytes.

The high frequency allelic variants of Hsp70s' in Kangri cancer, like Hsp70-homC/C and Hsp70-2 A/G or G/G haplotypes in our population, might express well, possibly by increased stability of variant mRNA or by altering the timing of co-translational folding that produce protein with altered enzymatic activity, so as to facilitate anti-apoptosis and delay senescence, and hence support tumor development. In our previous study, we reported *TP53* mutations in 32% of the sporadic Kangri cancer cases, therefore, an increase in expression of HSP genes is very likely, which further facilitates the various steps involved in tumor development.

The presence of high frequency genotypes of HSP70genes when compared with various clinical parameters of prognostic significance substantiates the role of Hsp70-2A/G or G/G genotype as risk imposing as evident from its presence in Kangri cancer cases bearing poor prognostic features. However, according to Manley's (2005) BADGE (Better Association for Disease and Gene) classification, the Hsp70-2gene-disease association is of 3^rd^-4^th^ class (based on *P* -values) that suggests low assurance of reproducibility.[25] In summary the high frequency of Hsp70-2A/G or G/G and Hsp70-homCC haplotype in Kangri cancer cases compared to controls suggest them as susceptibility genotypic variants for Kangri cancer to our population. Further, the significant association of high frequency allelic variants of HSP70genes in Kangri tumors with various clinico-pathological parameters suggests them as prognostic indicators as well. The high frequency, especially, of Hsp70-2A/G or G/G genotype in Kangri tumors of patients with such clinico-pathological features as advanced Clinical tumor stage [T2 (a,b)] and histopathological grade II, is a finding which assumes significance in view of the fact that these features reflect poor prognosis. The study suggests Hsp70-2A/G or G/G and Hsp70-homCC genotypes as attractive susceptibility markers and independent prognostic indicators in Kangri cancer patients of Kashmiri population. Nevertheless, these observations need further investigation, a larger cross section of the Kangri cancer patients and relevant control.
